# Concentric Circular Grating Generated by the Patterning Trapping of Nanoparticles in an Optofluidic Chip

**DOI:** 10.1038/srep32018

**Published:** 2016-08-23

**Authors:** Hailang Dai, Zhuangqi Cao, Yuxing Wang, Honggen Li, Minghuang Sang, Wen Yuan, Fan Chen, Xianfeng Chen

**Affiliations:** 1 The State Key Laboratory on Fiber Optic Local Area Communication Networks and Advanced Optical Communication Systems, Department of Physics and Astronomy, Shanghai JiaoTong University, Shanghai, 200240, China; 2 College of Physics & Communication Electronics, Jiangxi Normal University, Nanchang, Jiangxi, 330027, China; 3 Photonlabs Company, Shanghai, 200240, China

## Abstract

Due to the field enhancement effect of the hollow-core metal-cladded optical waveguide chip, massive nanoparticles in a solvent are effectively trapped via exciting ultrahigh order modes. A concentric ring structure of the trapped nanoparticles is obtained since the excited modes are omnidirectional at small incident angle. During the process of solvent evaporation, the nanoparticles remain well trapped since the excitation condition of the optical modes is still valid, and a concentric circular grating consisting of deposited nanoparticles can be produced by this approach. Experiments via scanning electron microscopy, atomic force microscopy and diffraction of a probe laser confirmed the above hypothesis. This technique provides an alternative strategy to enable effective trapping of dielectric particles with low-intensity nonfocused illumination, and a better understanding of the correlation between the guided modes in an optical waveguide and the nanoparticles in a solvent.

Optical micromanipulation, with its capability to non-invasively move or trap micro- or nano-objects, has attracted much attention in the past decade and contributed to the development of many fields, such as biology, colloidal dynamics, particle sorting, and lab-on-a-chip technology^1^. Several exciting developments have occurred recently. The optical trapping of small objects such as dielectric spheres^2^, carbon nanotubes^3^, semiconductor nanowires^4^, metal nanoparticles^5^,^6^,^7^ and even bio-particles^8^,^9^,^10^ has already been experimentally demonstrated^11^. Despite their different configurations, the key to realize effective optical trapping is to achieve a very strong electric field gradient, since the competition between the gradient and scattering force determines the trapping efficiency.

Among these trapping techniques, optical tweezers are widely used for various optical micromanipulation applications. Optical tweezers usually consist of a single-beam optical trap based on a standard microscope using a laser as its light source. The three-dimensional confinement can be achieved at the focal point through gradients. To achieve a tightly focused laser beam, high numerical aperture (NA) lenses are usually indispensable, and relatively high laser power is also required to obtain large gradients near the focal point. Consequently, the corresponding system is rather expensive. Near-field trap is another major type of optical-trapping technique based on the near-field optical schemes, such as whispering gallery mode-based micro-resonators^12^, plasmonic nano-antennas^13^, slot waveguides^14^ and photonic crystal cavities^15^. These methods can overcome the free space diffraction limit imposed by conventional optics, and trap particles whose sizes are smaller than the wavelength. Owing to the near-field enhancement effect in nano-scale structures, low incident power is enough to achieve effective trapping. However, to realize a near-field trap, rather complicated nanofabrication processes are required to produce micro- or nano-scale features. Furthermore, although substantial work has been conducted on the optical trapping issue, most has been focused on trapping and manipulating a single particle. Few research has been devoted on the issue of trapping many particles simultaneously, which may find wider application in particle stacking, sorting and filtering. K. Dholakia *et al*. performed pioneering work to demonstrate the optical trapping of spatially separated particles using both a single Bessel beam and a dual beam-interference optical lattice^16^. Such optical-trapping techniques^17^,^18^,^19^,^20^ can broaden the application of ‘lab-on-a-chip’ and the optical assembled microstructures. However the use of high NA lenses and high incident power hinders its application and further development. It is also relatively difficult to generate spatially sculptured beams^21^. Without an external optical projection lens, the optical lattice produced by an array of plasmonic antennas is applied to trap hundreds of micro-particles^22^. However, this technique still requires complicated nanofabrication of the plasmonic antenna array.

In this work, we take a different approach to trap multiple particles in an optofluidic chip. A hollow-core metal-cladded waveguide (HCMW) chip of millimeter-scale was used as a fluid-transport channel, and the unique ultrahigh-order modes (UOMs)^23^ are excited to realize the trapping of the nano-particles in the fluid. Since the UOMs at small incident angles are omnidirectional, a concentric ring pattern of the optical intensity distribution is formed in the sample cell near the incident spot. Due to the strong optical confinement of the waveguide structure, a large gradient force is obtained when the condition for UOMs resonance is fulfilled, which is the key factor of the effective optical trapping^24^,^25^,^26^,^27^. In our scheme, large quantities of nanoparticles can be trapped in a concentric circular pattern, which follows the field distribution of the UOMs. Compared with other approaches, our method does not require high NA lenses or high incident power of laser. Additionally, no complicated nano-fabrication process is involved. In the experiment, gold nanoparticles whose size distribution varies from 1 nm to 10 nm and follows normal distribution with central size is about 5.3 nm are used to verify the trapping mechanism. The nanoparticles were prepared by using polyvinyl pyrrolidone (PVP) as a stabilizer and then dispersed in deionized water, and its concentration is about 2.44 × 10^−7^ mol/ml. The nanoparticles can be efficiently trapped in the HCMW with the application of a low power laser. We also presented an experiment to demonstrate the concentric circular grating of the trapped nanoparticles by the diffraction pattern of a scattered beam. Direct observations via the scanning electron microscopy (SEM) and atomic force microscopy (AFM) are also carried out. This work presents a new strategy of simultaneously trapping a large amount of particles to form a regular pattern. Also a better understanding of the interaction between the optical guided modes and the nanoparticles in the optofluidic chip is provided.

## Results and Discussion

### Reflection cones of the UOMs

**Figure 1:**
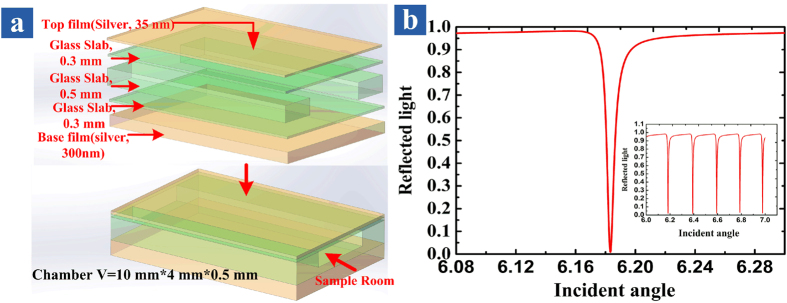
(**a**) Schematic diagram of the HCMW, where the analyte layer and two glass slabs serve as the guiding layer, which are sandwiched between two silver films, i.e., the coupling layer and the substrate. (**b**) Calculated reflectivity of the UOMs with respect to the incident angle; the simulation parameters are given in the text.

The first illustration is to show that the interaction between the UOMs and the morphology of the coupling layer of the HCMW chip can produce a series of reflection cones. In order to exploring the underlying physics, a brief introduction of the HCMW chip and the excited UOMs is necessary. The structure of HCMW is shown in Fig. 1(a), whose coupling layer is a 35 nm thick silver film deposited on a thin glass slab of 0.3 mm thick. Another 300 nm thick silver film serves as the substrate to prevent light leakage. The guiding layer is of 1.1 mm thick and consists of two glass slab and a sample room of 0.5 mm thick. Note that the volume of the cavity is about 0.02 ml.

**Figure 2:**
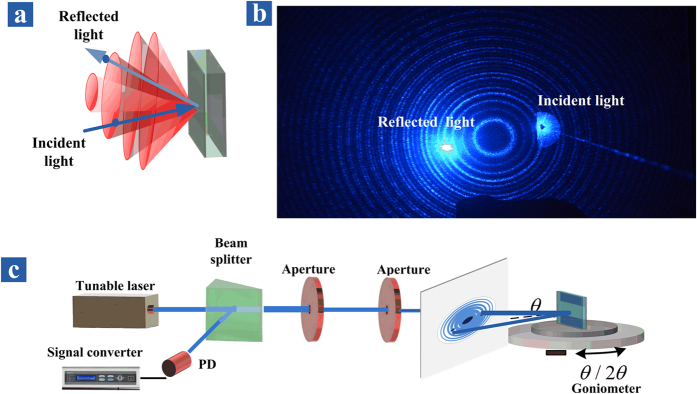
(**a**) A series of hollow cones of directionally scattered light, whilst the axis of the cones is normal to the HCMW chip surface. (**b**) Experimental results. (**c**) Schematic representation of the experimental setup.

Figure 1(b) plots a calculated reflectivity of the UOMs which can be excited by the free spacing coupling technology^28^,^29^, since the effective index of the HCMW chip is less than unit^30^,^31^,^32^. When the phase matching condition is fulfilled, energy is transferred from the reflected light and coupled into the guided UOMs, thus a reflection dip is formed in the reflection spectrum near the resonance angle and the optical intensity in the guiding layer is greatly enhanced compared to the incident light. Another important feature of the UOMs is that their mode density is high (as shown in the subplot of Fig. 1(b)), so the difference between the propagation constant of two adjacent modes is relatively small. A laser with small incident angle focused on the coupling layer can be scattered randomly by its natural roughness, and some of these scattered components have an appropriate wavevector to excite the corresponding UOMs, which propagate along the guiding layer. Furthermore the coupling between adjacent UOMs (inter-mode coupling) and the coupling between the same order mode with different propagation directions (intra-mode coupling) are possible, since the differences between their propagation constant are small. As a result, UOMs of different orders and propagation directions are excited via the two coupling mechanism mentioned above. When these UOMs are converted back to freely propagating light though the leakage radiation from the coupling layer, a set of bright ring patterns can be observed on a screen, while the specular reflected beam only produces a single spot. In short, incident energy can be coupled into and stored in the guiding layer; and since HCMW chip is a leakage waveguide, leakage radiation through each UOM produce a reflection cone. The experimental setup is shown in Fig. 2(c). A collimated beam from a 473 nm diode-pumped solid-state (DPSS) laser (Model TEM00, 25 mW) passes through a hole in the projection screen and hits onto the HCMW chip, which is placed on a step motor controlled *θ*/2*θ* goniometer. The sample room is empty and the experimental demonstration of the ring patterns on the screen is shown in Fig. 2(b). The physics behind is not difficult since a rather similar phenomenon is already demonstrated via the surface plasmon resonance (SPR), where only one hollow cone of directionally scattered light can be observed^33^. The experimental difference between the UOMs and the SPR can be easily understood since there is only one resonance dip in the SPR reflection spectrum.

### Patterning Trapping of Nanoparticles via UOMs

**Figure 3:**
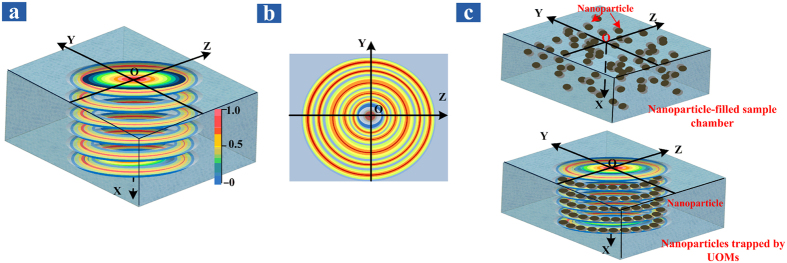
(**a**) Schematic illustration of the intensity distribution of the optical field inside the sample room. (**b**) The optical intensity distribution on the YZ plane. (**c**) Schematic diagram of the trapped nanoparticles by the UOMs.

Given the high intensity of the UOMs in the guiding layer^34^, it is natural to explore the possibility of the simultaneously trapping of massive nanoparticles in the aqueous environment. From the reflection cones experiment in Fig. 2, the distribution of the optical intensity as a consequence of the interaction between coupling layer morphology and UOMs properties in the sample room can be determined. As can be seen in Fig. 3(a), the optical intensity oscillates along the x direction, since the UOMs are similar to the Fabry-Perot mode due to the reflection at the two metallic interfaces. On the other hand, the UOMs are omnidirectional at small incident angle and different orders of UOMs have slightly different propagation constant. So in the yz-plane, the optical intensity in the radial direction is periodic and a concentric ring pattern as plotted in Fig. 3(b) is formed. When no UOMs are excited, the nanoparticles are supposed to distribute evenly in the solvent due to the Brownian motion effect. With the excitation of UOMs, the density of the trapped nanoparticles should be proportional to the local optical intensity, so the trapped nanoparticles will form a rather similar structure with the optical intensity distribution, as plotted in Fig. 3(c).

**Figure 4:**
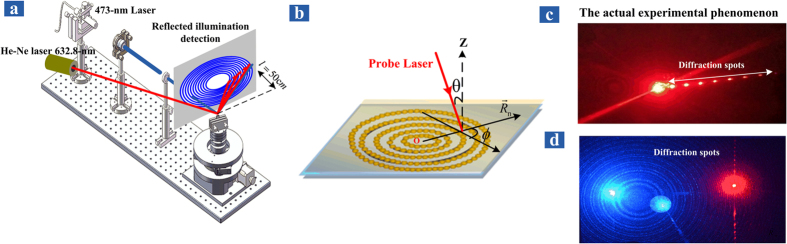
(**a**) Experimental setup of the diffraction of a 632.8 nm probe laser by the concentric circular grating, which consists of nanoparticles trapped by a 473 nm pump laser. (**b**) The details of the propagation direction of the 632.8 nm probe laser in a spherical coordinate system. The polar angle *θ* is measured from the normal direction of the chip surface at the incident position, while the azimuth angle *ϕ* is measured from the radial direction that passes through the center of the circular grating, i.e., the incident spot of the 473 nm pump laser. (**c**) Experimental diffraction pattern of the probe laser when the azimuth angle *ϕ* is about 90 degree. (**d**) Experimental diffraction pattern of the probe laser when the azimuth angle *ϕ* is about 5 degree.

Experimental prove must be provided to support the above hypothesis, and the experimental setup is shown in Fig. 4(a). In addition to the experiment of reflection cones, another He-Ne laser (632.8 nm, 5 mW), whose incident power is reduced by 80%, is also incident onto the HCMW chip with a different angle. Note that the 632.8 nm laser has a rather smaller power than the 473 nm laser, the influence of the 632.8 nm laser on the nanoparticles trapping can be ignored. Consequently, we will refer to the 473 nm laser as the pump laser and the 632.8 nm laser as the probe laser from now on. The details of the propagation direction and the incident spot of the probe laser is plotted in Fig. 4(b), and note that the incident spots of the pump and probe lasers don’t completely coincide. Two typical experimental results are shown in Fig. 4(c,d) with different azimuth angle *ϕ*. It is clear that the diffraction direction changes correspondingly as the azimuth angle *ϕ* of the probe laser varies. This is a strong evidence of the circular pattern of the trapped nanoparticles, because a rather simple demonstration can be easily done with a cheap semiconductor laser and a DVD disk.

### Concentric circular grating of the deposited nanoparticles

**Figure 5:**
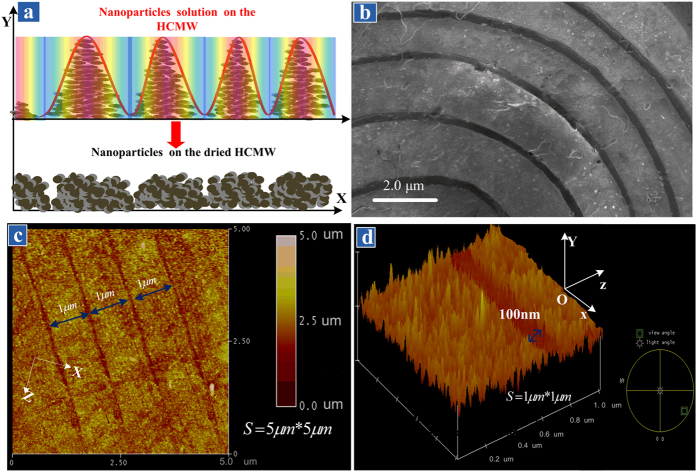
(**a**) SEM image of the concentric distribution of grooves formed by deposited nanoparticles on the sample room floor. (**b**) Details of five concentric grooves close to the center, whilst the spacing between two grooves is of micrometer scale. (**c**) AFM image of the deposited nanoparticle grating near the edge in a 5*μm* × 5*μm* area, whose period is smaller in comparison of that plotted in Fig. 5(b). (**d**) Details of a single groove in a 1*μm* × 1*μm* area shows it is roughly 100 nm wide.

The SEM and AFM can provide more direct evidence than the above diffraction experiment, but the trapped particles need to be deposited without completely destroying the pattern. So the solvent inside the sample room is gradually evaporated while the pump laser is on and the other experimental condition remains unchanged. The room temperature is 23 degree and the whole evaporation process takes about 10 minutes. Afterward, the chip was dried in drying oven (CS 101-1E) for 6 hours at 40 °C. It should point out that although the optical intensity distribution in the sample room varies gradually as the volume of liquid is decreasing; it always takes the same structure as shown in Fig. 3(a). In other words, the intensity distribution in the yz-plane remains in the form of concentric ring pattern, because the UOMs propagates along the guiding layer in all azimuthal directions with different propagation constant. Thus the concentric circular pattern of the trapped nanoparticles will always exist throughout the evaporation process. When the evaporation is completed, the upper 0.3 mm glass slab and the coupling layer are removed, and direct observations can be performed to investigate the pattern formed by the deposited nanoparticles. The results are shown in Fig. 5, where a concentric rings pattern of the deposited particles is about 100 um in diameter. The spacing between two adjacent grooves is larger in the center region than the edge, this is due to the fact that the difference of the propagation constant of the higher order modes is larger. All topography and surface roughness data were acquired using AFM in a non-vibrational mode at room temperature. The topography area of 5 μm × 5 μm was constructed using 512 scan lines and scan rate of 2.441 Hz. Since the nanoparticle pattern is much related with the optical intensity field, it can be conclude that the field intensity distribution in the yz plane in the guiding layer has a similar form as that of the observed reflection cones on the screen. Figure 5 also shows the grating period is of micrometer scale and the groove width near the edge is about 100 nm.

**Figure 6:**
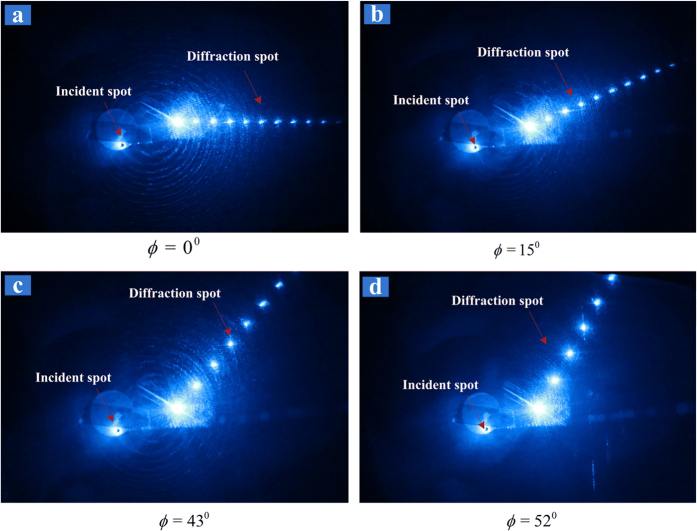
The diffraction pattern of a 473 nm probe laser at different azimuth angle *ϕ* when incident directly on the deposited nanoparticle grating, which is constructed via the excitation of the UOMs and the solvent evaportation. From (**a**) to **(d**), the azimuth angle *ϕ* are 0°, 15°, 43° and 52°, respectively; while the polar angle *θ* is fixed at 20 degree.

After removing the upper glass slab and the coupling layer, the remained structure includes the deposited particle layer, the lower glass slab and the metal substrate. The deposited nanoparticles can be used to diffract probe laser directly, while the pump laser is no longer required. Figure 6 shows the diffraction experiment of the 473 nm laser with different azimuth angle *ϕ*. It can be seen that the distance between the diffraction spots increases as the azimuth angle becomes larger, and the diffraction direction also varies. Careful readers may notice that some ring patterns on the screen are still discernible, while the original HCMW structure is already destroyed. The reason can be explained as follows: the lower glass slab is still 0.3 mm thick, which is thick enough to hold UOMs, and the circular grating of deposited particles acts as grating coupler to excite these modes. While the metal substrate prevents leakage from the bottom, the reflection cones can be observed as the leakage radiation from above. So the experimental result in Fig. 6 is different from that in Fig. 4, where both reflection cones and diffraction pattern can be observed via a single laser beam. In the last illustration, the deposited circular grating generates the diffraction pattern, whilst the new grating-coupled waveguide structure leads to the reflection cones.

## Conclusions

We analyze the mechanism of the inter- and intra-mode coupling of the UOMs excited in the HCMW chip, which results in a series of reflection cones. The optical intensity inside the sample room of the waveguide chip is circularly distributed in the yz-plane (as shown in Fig. 3), and oscillates along the x axis. The possibility of simultaneously trapping of massive nanoparticles in a solvent via the optical fields of the UOMs is verified via the diffraction phenomenon of a probe laser. Since the intensity distribution of the UOMs has a similar spatial structure as the solvent is gradually evaporated, a concentric circular grating of the deposited nanoparticles can be obtained. Both SEM and AFM observations and the diffraction experiment provide concrete evidences of the above hypothesis. The potential application of this work includes the manipulation of the massive trapped particles via adjusting the waveguide structure or the laser parameters, e.g., its incident angle, wavelength and polarization. The related study would be of great interest to various fields such as optofluidics, advance material synthesis and biomedical research.

## Additional Information

**How to cite this article**: Dai, H. *et al*. Concentric Circular Grating Generated by the Patterning Trapping of Nanoparticles in an Optofluidic Chip. *Sci. Rep.* **6**, 32018; doi: 10.1038/srep32018 (2016).
